# Importance of All Movement Behaviors in a 24 Hour Period for Overall Health

**DOI:** 10.3390/ijerph111212575

**Published:** 2014-12-04

**Authors:** Jean-Philippe Chaput, Valerie Carson, Casey E. Gray, Mark S. Tremblay

**Affiliations:** 1 Healthy Active Living and Obesity Research Group, Children’s Hospital of Eastern Ontario Research Institute, 401 Smyth Road, Ottawa, ON K1H 8L1, Canada; E-Mails: casgray@cheo.on.ca (C.E.G.); mtremblay@cheo.on.ca (M.S.T.); 2 Faculty of Physical Education and Recreation, W1-34 Van Vliet Centre, University of Alberta, Edmonton, AL T6G 2H9, Canada; E-Mail: vlcarson@ualberta.ca

**Keywords:** sleep, sedentary behavior, physical activity, exercise, health, children

## Abstract

Physical inactivity and childhood obesity are well-recognized public health concerns that are associated with a range of adverse health outcomes. Historically, the benefits of physical activity (e.g., moderate-to-vigorous physical activity—MVPA) to overall health have dominated discussions and emerging evidence indicates that a broader, more integrated approach is needed to better understand and address current public health crises. Existing guidelines for children and youth around the world only focus on MVPA, and recently sedentary behavior, despite an accumulating body of evidence showing that light-intensity physical activity (LPA) such as walking can provide important health benefits. Furthermore, there is accumulating support for the importance of adequate sleep and that these behaviors moderate the health impact of each other. Ignoring the other components of the movement continuum (*i.e.*, sleep, sedentary time, LPA) while focusing efforts exclusively on MVPA (accounting for <5% of the time in a 24 h period) limits the potential to optimize the health benefits of movement behaviors. In order to address this limitation, experts in Canada are currently developing the world’s first *Integrated 24 Hour Movement Behaviour Guidelines for Children and Youth* to help advance an integrated healthy active living agenda that has the potential to significantly improve the overall health and well-being of children and youth.

## 1. Introduction

Human movement has been a necessity for survival throughout evolution. However, sitting has become the new norm in today’s environment despite the fact that we have not genetically adapted to this sedentary lifestyle [1]. It is now common to refer to physical activity as having “health benefits”, even though the active state is the normal biological condition for metabolic processes [2,3]. Lack of human movement should rather be perceived as “abnormal” and associated with numerous health risks [4,5].

Physical inactivity and childhood obesity represent pervasive, and arguably the greatest, health challenges to our children today [6,7]. There is no easy solution to these serious public health threats and they need to be addressed by a range of strategies to maximize success. One of the strategies is to find ways to increase overall physical activity of children. However, health research has mainly focused on the impact of moderate-to-vigorous physical activity (MVPA), or even high-intensity physical activity, on various health outcomes. Although this approach has resulted in important contributions to the field, emerging evidence indicates that a broader, more inclusive and more integrated approach to understanding and promoting human movement is required to better address the current public health crisis of physical inactivity and childhood obesity.

**Figure 1:**
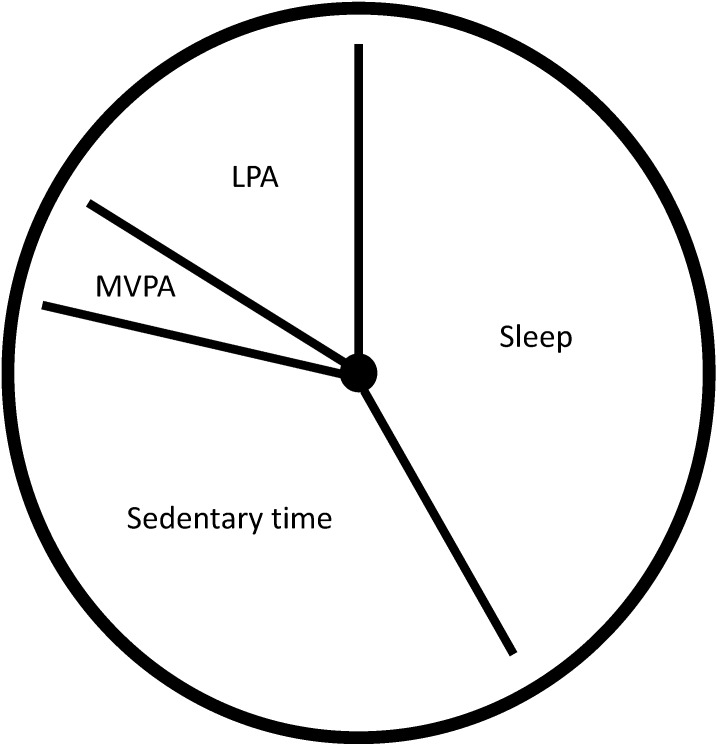
Estimated distribution of movement behaviors over the 24 h period.

LPA, light-intensity physical activity; MVPA, moderate-to-vigorous physical activity.

There is no doubt that MVPA provides many important health benefits. However, MVPA only accounts for a small proportion (<5%) of the 24 h day, even among active children and youth. In contrast, sleep (~40%), sedentary behavior (~40%) and LPA (~15%) make up approximately 95% of the day (Figure 1). Ignoring other components of the movement continuum while focusing efforts on MVPA limits our understanding of how habitual movement behaviors interact to impact children’s health. Indeed, recent efforts to address the single-minded focus on MVPA have led to a proliferation of sedentary behavior research and to the creation of the world’s first sedentary behavior guidelines for children and youth [8,9,10].

There is accumulating evidence that excessive sedentary behavior, particularly screen-based sedentary behavior (*e.g.*, television viewing), has unfavorable effects on various health indicators, independent of MVPA [8,9]. Likewise, short sleep duration is associated with many adverse health outcomes including obesity, type 2 diabetes, depression, suicidal ideation, and poor academic performance [11,12,13,14]. This issue is of particular concern since a decrease in sleep duration has been observed over the past decades in children and adolescents [15,16]. Finally, a growing body of evidence indicates that spending more waking hours in LPA compared to sedentary pursuits can provide health benefits [17,18,19]. Reallocating sedentary time to LPA (*e.g.*, TV watching to active play) is of substantial public health interest given that it is not feasible to participate in MVPA during all waking hours. Breaking up sedentary time with LPA is a more achievable and viable goal for increasing movement (*i.e.*, number of steps per day) and acquiring associated health benefits than focusing solely on MVPA. However, current physical activity guidelines only focus on MVPA (and recently sedentary behavior) and do not take into consideration other important movement/non-movement behaviors that occur throughout the day. This issue is important because having one “unhealthy” movement behavior can moderate the health benefits of another. For example, the health benefits of MVPA can be mitigated if children have poor sleep habits and/or engage in excessive sedentary behavior [20]. Conversely, increased physical activity could well decrease the detrimental effects of insufficient sleep and/or extended periods of sitting in some individuals [21,22].

## 2. Is There a Need for 24 Hour Movement Behavior Guidelines?

**Figure 2:**
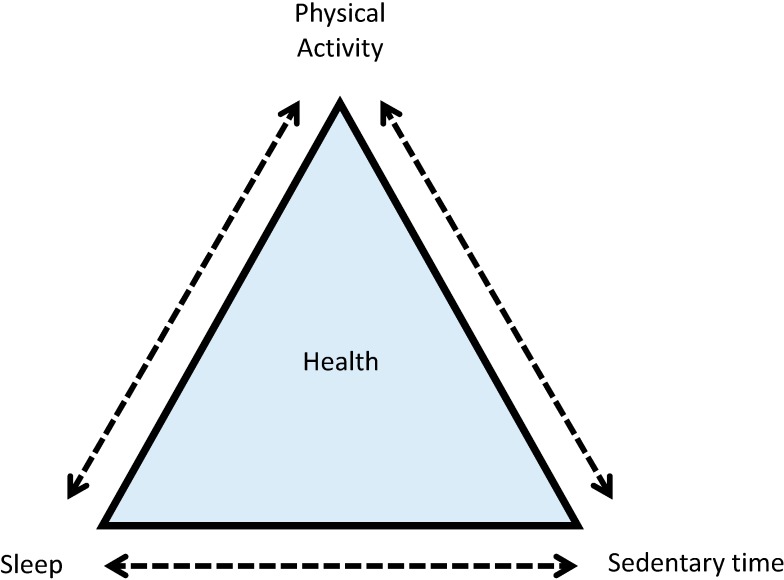
Interactions between movement and non-movement behaviors to collectively impact health.

Within a 24 h period, movement occurs on a continuum from sleep (*i.e.*, no/low movement) to vigorous-intensity physical activity (*i.e.*, high movement). However, current guidelines are missing information on at least two important movement behaviors: LPA and sleep. Ignoring important components of the 24 h day is unfortunate as new research shows that all movement behaviors and how they interact in a 24 h period have important health implications [23,24]. A body of behavior change research indicates that an integrated or holistic approach, which incorporates all movement behaviors, will result in a larger impact on health indicators compared to an approach that only focuses on single behaviors [25]. Based on Rogers’ diffusion of innovations theory [26], integrated guidelines are more likely to be adopted if they are perceived to offer a relative advantage over individual guidelines. Indeed, preliminary work by our group indicates that less than 40% of pediatricians surveyed across Canada reported they almost always make physical activity and sedentary behavior recommendations to parents/caregivers of children and youth at regular checkups [27], whereas 76% stated they would be more likely to counsel patients on physical activity, sedentary behavior and sleep if one integrated guideline document outlining recommendations for all these behaviors existed. Given the current inactivity and obesity crisis, a new approach that can have a larger impact over our current approaches is desperately needed. Therefore, it is time that we adopt a paradigm that integrates, not segregates, movement behaviors (Figure 2).

In order to address this issue, experts in Canada are currently developing the world’s first *Integrated 24 Hour Movement Behaviour Guidelines for Children and Youth* (aged 5–17 years). These guidelines will include all intensities of physical activity (light, moderate, vigorous), sedentary behavior, and sleep. They also follow established protocols for clinical practice guideline development [28], and involve a large team of researchers, knowledge users, and international collaborators. These new guidelines will help children, youth, parents, educators, public health/health care professionals, and governments easily understand the importance of all movement behaviors in a 24 h period. This evidence-informed approach will help to advance an integrated healthy active living agenda that has the potential to significantly contribute to the improvement of overall health and well-being among children and youth in Canada and worldwide.

## 3. Conclusions

The clustering and interactions among movement and non-movement behaviors suggest that all components of the 24 h movement continuum should be targeted to maximize health benefits. Participating in regular MVPA and minimizing sedentary behaviors (especially screen time) have been shown to provide numerous important health benefits [4,5,8,9]. In a similar manner, having healthy sleep hygiene and reallocating sedentary time to LPA have also been reported to provide valuable positive health benefits [13,14,17,19]. However, no recommendations or guidelines exist at present that follow an integrated approach including all components of the 24 h day. We believe that it is time to adopt a more holistic approach in this field of research and hope that other jurisdictions around the world will also follow this direction.
